# Surgical nonunion treatment of large-sized defects of femur and tibia based on the diamond concept

**DOI:** 10.1302/2633-1462.61.BJO-2024-0096.R1

**Published:** 2025-01-06

**Authors:** Sebastian Findeisen, Louis Mennerat, Thomas Ferbert, Lars Helbig, Tim N. Bewersdorf, Tobias Großner, Christian Schamberger, Gerhard Schmidmaier, Michael Tanner

**Affiliations:** 1 Clinic for Trauma and Reconstructive Surgery, Centre for Orthopedics, Trauma Surgery, and Paraplegiology, Heidelberg, Germany

**Keywords:** Nonunion, Nonunion therapy, Segmental bone defect, Masquelet technique, Diamond concept, fracture nonunions, femur, tibia, surgical treatment, Trauma Surgery, bone defects, radiological outcome, callus formation, open fractures

## Abstract

**Aims:**

The aim of this study was to evaluate the radiological outcome of patients with large bone defects in the femur and tibia who were treated according to the guidelines of the diamond concept in our department (Centre for Orthopedics, Trauma Surgery, and Paraplegiology).

**Methods:**

The following retrospective, descriptive analysis consists of patients treated in our department between January 2010 and December 2021. In total, 628 patients were registered, of whom 108 presented with a large-sized defect (≥ 5 cm). A total of 70 patients met the inclusion criteria. The primary endpoint was radiological consolidation of nonunions after one and two years via a modified Lane-Sandhu Score, including only radiological parameters.

**Results:**

The mean defect size was 6.77 cm (SD 1.86), with the largest defect being 12.6 cm. Within two years after surgical treatment, 45 patients (64.3%) presented consolidation of the previous nonunion. After one year, six patients (8.6%) showed complete consolidation and 23 patients (32.9%) showed a considerable callus formation, whereas 41 patients (58.6%) showed a Lane-Sandhu score of 2 or below. Two years after surgery, 24 patients (34.3%) were categorized as Lane-Sandhu score 4, another 23 patients (32.9%) reached a score of 3, while 14 patients (20.0%) remained without final consolidation (score ≤ 2). A total of nine patients (12.9%) missed the two-year follow-up. The mean follow-up was 44.40 months (SD 32.00). The mean time period from nonunion surgery to consolidation was 16.42 months (SD 9.73)

**Conclusion:**

Patients with presentation of a large-sized nonunion require a structured and sufficiently long follow-up to secure the consolidation of the former nonunion. Furthermore, a follow-up of at least two years is required in order to declare a nonunion as consolidated, given that a significant part of the nonunions declared as not consolidated at one year showed consolidation within the second year. Moreover, the proven “gold standard” of a two-step procedure, so called Masquelet technique, shows effectiveness.

Cite this article: *Bone Jt Open* 2024;6(1):26–34.

## Introduction

Managing fracture nonunions remains challenging in trauma surgery, especially when it comes to large-sized defects (≥ 5 cm). A correct consensus on the definition of a nonunion is still lacking. Until today, there is no unified definition with regard to standardized nonunion diagnosis. For example, the US Food and Drugs Administration (FDA) describes a nonunion as a fracture that shows inadequate osseous healing after nine months without any radiological signs of progression for at least three consecutive months.^1^ In our department (Centre for Orthopedics, Trauma Surgery, and Paraplegiology), we define a nonunion the same as the European Society of Tissue Regeneration in Orthopaedics and Traumatology (ESTROT),^2^ according to whom a nonunion is defined as a fracture that cannot heal without any further intervention, regardless the previous treatment duration.^3^

In general, the prevalence of nonunion varies between 5% to 10%, and can rise up to 30% in presence of certain risk factors.^1^ These can be patient-related, such as age, smoking status, diabetes, vascularization disorders or osteoporosis, or independent general risk factors, such as open fractures, defect size, local infection, and soft-tissue status.^4,5^

Patients with presentation of a nonunion who underwent surgical treatment at our department received a standardized treatment according to the so-called diamond concept, established throughout the last 20 years, and first published by Giannoudis et al^6^ in 2007. Initially, this concept consisted of four elements whose consideration is fundamental for fracture healing with osseous regeneration: addition of growth factors, use of osteoconductive scaffolds, addition of mesenchymal stem cells, and achievement of mechanical stability.^6,7^ Consecutively, a fifth element had been added by identifying the improvement of vascularization as further recommended condition for optimal fracture healing.^8^ Therefore, treatment by means of a two-step procedure with an induced membrane technique became the preferred option for surgical treatment, primarily of atrophic, infected, and large-sized nonunions.^9^

Because large-sized defects remain a rare complication in trauma surgery, resulting in a small number of treated patients, robust studies are lacking and so are the impacts of treatment options.

## Methods

### Study design

**Fig. 1:**
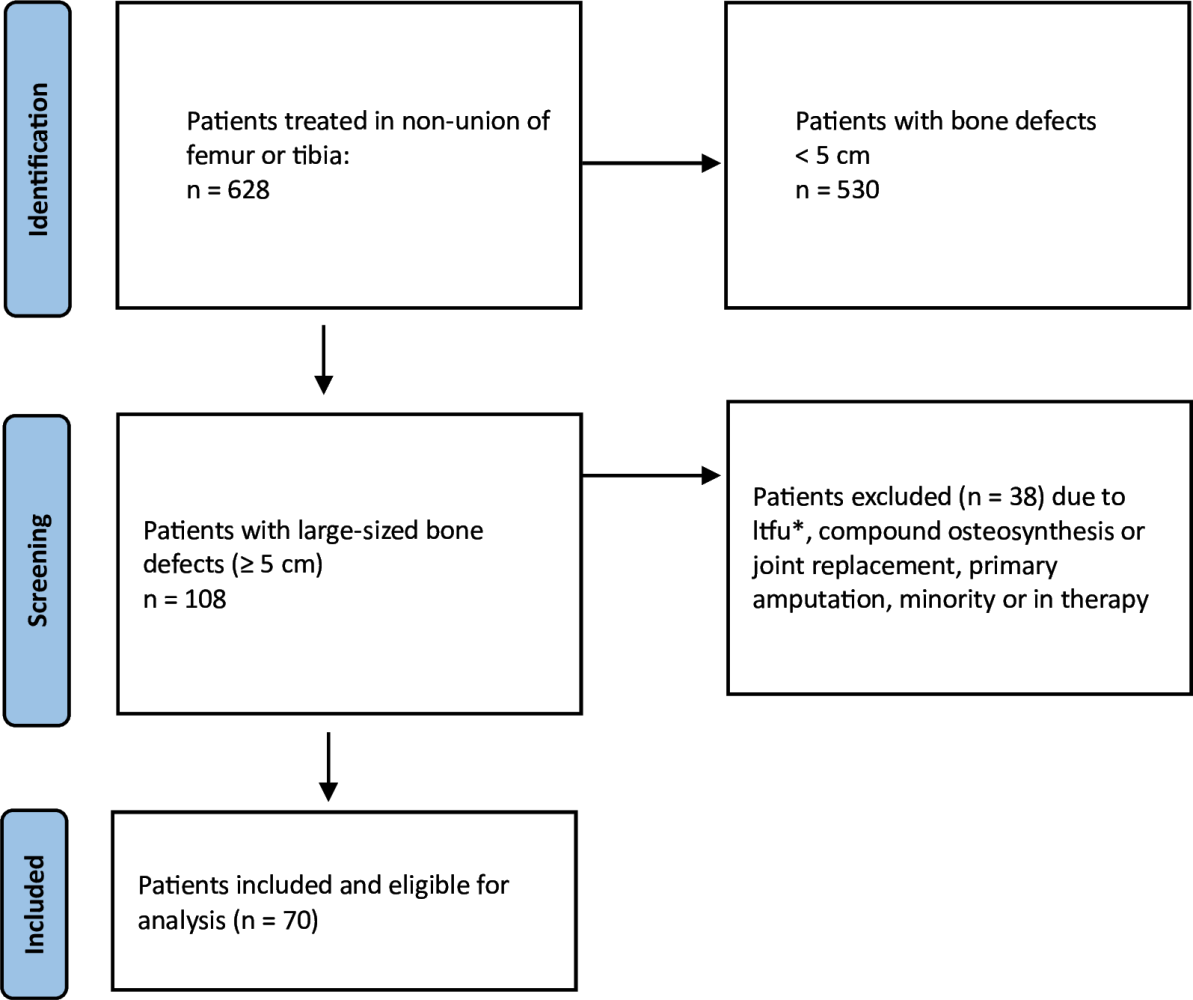
Visualizing the patient selection process. ltfu, loss to follow-up.

The following study was designed as a retrospective, descriptive database analysis. The analyzed data were collected out of our clinical nonunion database. The local ethics committee granted ethical approval (S-262/2017) and the study was conducted in accordance with the Declaration of Helsinki. The study consists of a database of nonunions presenting from January 2010 to December 2021. In this period, 628 patients with a nonunion of the femur or tibia were treated, of whom 108 presented with a large-sized defect (≥ 5 cm). After screening whether patients met the inclusion criteria, 38 were excluded for reasons such as loss to follow-up (LTFU) or non-biological reconstruction. The exact screening process can be seen in Figure 1.

The primary endpoint was radiological consolidation of the nonunion one and two years after surgical treatment. Secondary endpoints were the occurrence of complications or effects of risk factors on consolidation. To objectify bone stabilization after one and two years we employed a modified Lane-Sandhu score^10^ (Table I), which only included radiological parameters. A minimum score of 3 implies an adequate stability of the particular bone, whether a score of 4 implies a completed bone consolidation. A score of 2 or below is equivalent to inadequate stability and the nonunion is considered non-consolidated.^11^

**Table I.** Radiological findings of the modified Lane-Sandhu score.

Score	Radiological findings
0	No callus
1	Minimal callus
2	Callus evident, but healing incomplete
3	Callus evident with stability expected
4	Complete healing with bone remodelling

Overall, six patients underwent a single-step treatment, whereas 64 were treated via a two-step procedure (Masquelet technique).^12^

### Statistical analysis

For data analysis, the Statistical Program for Social Science (SPSS) v. 29.0 (IBM, USA) was used. All quantitative data were expressed as mean (SD) and median (IQR). All qualitative data were expressed as absolute count and by relative rate in percentage.

### Objectives

The aims and objectives of the following study were to analyze the radiological outcome of large-sized defects under treatment after the diamond concept, and whether risk factors might induce the development of those kind of defects. Furthermore, we want to determine noticeable data points as a basis for future studies.

### Surgical technique

**Fig. 2:**
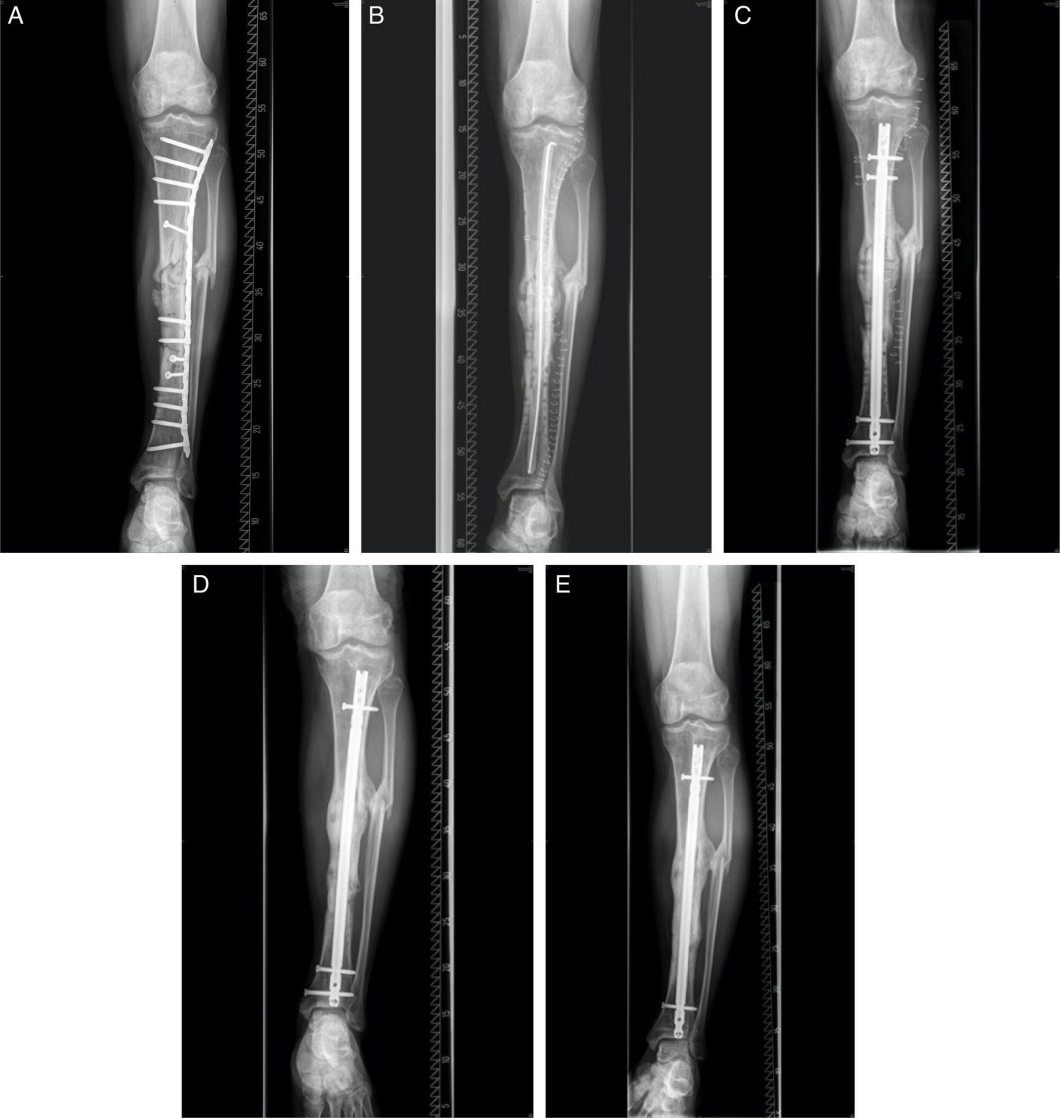
Radiological example for nonunion treatment under application of the diamond concept in our department with osseus consolidation. a) Radiological imaging (anteroposterior) before a nonunion treatment of an infected diaphyseal tibia nonunion (defect size ~13 cm) of a 60-year-old male patient. b) First surgical treatment occurred via Masquelet stage 1 with removal of previous implanted material, radical debridement, and implantation of a local and intramedullary antibiotic-laced polymethylmethacrylate (PMMA) spacer. c) After reaching clean septic status, Masquelet stage 2 was performed, removing the spacer and filling the induced membrane with autologous bone using reamer irrigator aspiration technique under further addition of Vitoss and implantation of a tibia nail, creating stability. d) At one-year follow-up, after dynamization of the nail, full osseus consolidation was achieved (Lane-Sandhu score 4). e) At two-years follow-up, further bone remodelling had occurred.

All patients were treated in accordance with previous publications from our department. Bone graft was mixed with either tricalcium phosphate (Vitoss, USA), bioactive glass (Bioglass; Bonalive Biomaterials, Finland), or a combination of both (Vitoss-BA; Vitoss). Optionally, growth factors were added (rhBMP-2 or rhBMP-7). A radiological example for nonunion treatment with osseous consolidation is shown below (Figure 2).

### Postoperative follow-up

All patients undergoing surgical treatment were included in this follow-up. At six weeks, three and six months, as well as one and two years after surgery, patients underwent clinical and radiological examination at our hospital. Clinical examination and radiological status (x-rays and/or CT scan) at one- or two-year follow-up were noted. Nonunion was declared consolidated with a Lane-Sandhu score of 3 or 4. All collected data were registered in our database for further statistical analysis.

## Results

### Patient characteristics

The majority of the treated patients were male (50; 71.4%) and 20 were female (28.6%). The mean age was 58.64 years (SD 13.49), with the youngest treated patient being aged 24 years and the oldest 88 years. In all, 23 out of 70 nonunions were located in the femur (32.8%), whereas 47 were located in the tibia (67.2%). The exact anatomical localization is evident from Table II.

**Table II.** Patient characteristics.

Variable	Data
**Sex, n (%)**	
Female	20 (28.6)
Male	50 (71.4)
Mean age, yrs (SD)	58.64 (13.49)
Mean bone defect before surgery, cm (SD)	6.77 (1.86)
**Affected region, n (%)**	
Femur proximal	5 (7.1)
Femur diaphyseal	10 (14.3)
Femur distal	8 (11.4)
Tibia proximal	9 (12.9)
Tibia diaphyseal	14 (20.0)
Tibia distal	24 (34.3)
**Nonunion treatment, n (%)**	
One-step	6 (8.6)
Two-step	64 (91.4)
Mean number of previous surgeries (SD)	6.25 (7.44)
**Performed osteosynthesis, n (%)**	
Intramedullary nail	32 (45.7)
Osteosynthesis plate	36 (51.4)
No change	2 (2.9)
**Bone substitute, n (%)**	
Vitoss	44 (92.9)
Bioglass	9 (12.9)
Vitoss-BA	9 (12.9)
None	8 (11.4)
**Smoking status, n (%)**	
Non smoker	35 (50.0)
Previous smoker	13 (18.6)
Smoker	22 (31.4)
**Type of fracture, n (%)**	
Closed	33 (47.1)
Open	37 (52.9)
Diabetes mellitus, n (%)	
No	60 (85.7)
Yes	10 (14.3)
**ASA grade, n (%)**	
1	16 (22.9)
2	47 (67.1)
3	6 (8.6)
4	1 (1.4)
**Clinical infection status, n (%)**	
Sterile	22 (31.4)
Previously infected or suspicion of infection	9 (12.9)
Septic	39 (55.7)
**Alcohol abuse, n (%)**	
No	60 (85.7)
Yes	10 (14.3)
**Drug abuse, n (%)**	
No	64 (91.4)
Yes	6 (8.6)
Mean BMI, kg/m^2^ (SD)	27.38 (5.52)
**Adiposity, n (%)**	
Adiposity permagna	16 (22.9)
No adiposity permagna	54 (77.1)
**Consolidation, n (%)**	
Yes	45 (64.3)
No	25 (35.7)

ASA, American Society of Anesthesiologists.

Every patient treated presented with a large-sized defect of minimum 5 cm. The mean defect size was 6.77 cm (SD 1.86), with the largest defect being 12.6 cm. In order of previous treatment options, six patients out of 70 received a one-step nonunion surgery (8.6%), whereas 64 patients (91.4%) were treated with a two-step treatment. The mean number of previous surgeries of the nonunion before presenting at our department was 6.25 (SD 7.44), with the highest number of previous surgeries being 40. Overall, 33 patients (47.1%) presented with a closed fracture, and 37 (52.9%) with an open fracture. By subdividing the group of open fractures into grade 1 to 3 according to Tscherne and Oestern,^13^ four patients presented with an “open 1°” (5.7%), 16 with “open 2°” (22.9%), and 17 with an “open 3°” fracture (24.3%). Patient-related and general risk factors are illustrated in Table II.

### Outcome after treatment and follow-up

A total of nine patients (12.9%) received Bioglass, 44 (62.9%) Vitoss, nine (12.9%) Vitoss-BA, and eight (11.4%) solely autologous bone. In all, 32 patients (45.7%) received an intramedullary nail, 36 (51.4%) a plate osteosynthesis, whereas two patients (2.9%) received no new implant. In total, patients presented with a mean number of 10.96 (SD 9.74) surgeries prior. Overall, 43 patients (38.6%) underwent at least one revision surgery (61.4%), and 27 indicated no need of revision. Reasons for revision are shown in Table III. The mean follow-up was 44.40 months (SD 32.00). Patients who did not reach full consolidation after two years had a mean follow-up period of 53.92 months (SD 31.82), whereas patients with consolidation of the nonunion had a mean follow-up of 39.11 months (SD 31.21). Regarding the period of nonunion treatment to consolidation, the mean was 16.82 months (SD 9.49). The mean time from initial trauma to consolidation was 45.56 months (SD 72.43), the longest being 499 months (Table IV).

**Table III.** Reasons for revision surgeries.

Reason	N (%)
Debridement	21 (30)
Implant removal	17 (24.3)
Implant material failure	14 (20)
Correction of implanted material	9 (12.9)
Excision of fistula	7 (10)
Wound healing disorder	6 (8.6)
Prosthesis	5 (7.1)
Amputation	4 (5.7)
Dynamization	3 (4.3)
Compound osteosynthesis	3 (4,3)
Arthrolysis	1 (1,4)
Persistent osteitis	1 (1,4)
Osteomyelitis	1 (1,4)
Arthrodesis	1 (1,4)

**Table IV.** Follow-up in relation to consolidation.

Variable	No		Yes	
	**Mean (SD)**	**Median (IQR)**	**Mean (SD)**	**Median (IQR)**
Follow-up, mths	53.92 (31.82)	48 (37; 35 to 72)	39.11 (31.21)	28 (32; 18 to 50)
Nonunion treatment to consolidation, mths			16.82 (9.49)	14 (9; 11 to 20)
Trauma to consolidation, mths			45.56 (72.43)	32 (18; 23 to 41)

In review of radiological findings at one-year follow-up, one patient (1.4%) showed no callus formation, and 20 patients developed minimal callus or callus with incomplete healing (each 28.6%). Another 23 patients (32.9%) presented callus with adequate stability, whereas six patients (8.6%) achieved full osseous consolidation (Table V).

**Table V.** Lane-Sandhu score one year after treatment.

Score	N (%)
0 (no callus)	1 (1.4)
1 (minimal callus)	20 (28.6)
2 (callus evident but healing incomplete)	20 (28.6)
3 (callus evident with stability expected)	23 (32.9)
4 (complete healing with bone remodelling)	6 (8.6)
Total	70 (100.0)

At two years’ follow-up, ten patients remained at Lane-Sandhu score 1 (14.3%), and four patients presented evident callus but without complete healing (5.7%). A further 23 patients showed callus with stability expected (32.9%) and 24 patients (34.3%) were categorized as complete healing with full osseous consolidation. Overall, nine patients (12.9%) missed out on the two-year follow-up (Table VI).

**Table VI.** Lane-Sandhu score two years after treatment.

Score	N (%)
0 (no callus)	0 (0.0)
1 (minimal callus)	10 (14.3)
2 (callus evident but healing incomplete)	4 (5.7)
3 (callus evident with stability expected)	23 (32.9)
4 (complete healing with bone remodelling)	24 (34.3)
Missing (no two-year follow-up)	9 (12.9)
Total	70 (100.0)

### Risk factors and comorbidities

Of the 70 patients included in this study, 22 (31.4%) were actively smoking. Out of the smokers, 13 patients (59.1%) reached full consolidation after two years, whereas nine patients (40.9%) showed inadequate consolidation. Of the non-smokers, ten patients (28.6%) did not reach consolidation status, and 25 (71.4%) presented adequate stability, thus consolidation of the nonunion. With regard to presentation of adiposity, 16 patients (22.9%) were categorized as patient with adipositas permagna, of whom seven (43.8%) consolidated, whereas nine patients (56.3%) did not reach full consolidation. The remaining 54 patients (77.1%) who were of regular weight showed a consolidation rate of 70.4% (equal to 38 patients), toward 29.6% without final consolidation (equal to 16 patients). All other comorbidities are shown in Table VII.

**Table VII.** Risk factors in relation to consolidation.

Variable	No	Yes
**Smoking status, n (%)**		
Non smoker	10 (14.3)	25 (35.7)
Previous smoker	6 (8.6)	7 (10.0)
Smoker	9 (12.6)	13 (18.6)
Infection during nonunion therapy, n (%)	49 (30.0)	21 (70.0)
Open fracture, n (%)	32 (45.7)	38 (54.3)
Diabetes mellitus, n (%)	25 (35.7)	45 (64.3)
Achohol abuse, n (%)	27 (38.6)	43 (61.4)
Adipositas permagna, n (%)	47 (67.1)	23 (32.9)
Mean BMI, kg/m^2^ (SD)	30.02 (7.19)	25.97 (3.76)
Mean age, yrs (SD)	59.76 (12.56)	58.02 (14.07)

## Discussion

The aim of this study was to analyze the outcome of patients with large-sized nonunions of the long bones of the lower extremity, who were surgically treated according to the established diamond concept, and furthermore, to investigate whether certain risk factors might contribute to the development of this complication following a previous fracture. Furthermore, the effect of the diamond concept applied to large-sized-defects of femur and tibia was to be noted.

Considering the localization of nonunions, the tibia has shown to be the most concerning localization in the human body with an average incidence of 8.7%. Nonunions are most prevalent in the tibia at 45%, while the femur is affected in 16%.^3^ In accordance with this frequency distribution, in our collective, two out of three patients presented a large-sized defect of the tibia (67.2%). Patients presenting tibial nonunions were found to have a significantly higher rate of postoperative infections, probably due to more frequent open fractures and the poor soft-tissue coverage.^14^ In addition to the higher infection rate, patients with tibial defects under induced membrane treatment seem to have a lower union rate, as shown by Morris et al^15^ with a consolidation rate of 41.67%.

The results regarding the consolidation rate (64.3% of treated patients consolidated vs 35.7% without therapeutic success) were in consensus with prior studies analyzing consolidation after induced membrane technique.^16,17^ For large-sized defects, our results showed no significant difference to consolidation rates for smaller defects.

Patients with large-sized bone defects demonstrated a mean of 16.42 months (SD 9.73) to consolidation, according to our analysis. This shows, in unison with other study results, that a follow-up of one year is insufficient for evaluation of the final consolidation. The period of follow-up should be prolonged to at least two years after surgical treatment. Prior publications referred to lower times to healing; this might be caused by the smaller average sizes of defects. However, Shen et al^18^ published a systematic review in which they analyzed patients with infected bone defects treated with the induced membrane technique. Although the results were among the same defect size (mean defect 6.8 cm (0.5 to 30)), the mean time to healing was significantly shorter at 7.5 months (2.3 to 49.9). Another observational study analyzing large septic nonunions of the femur and tibia after induced membrane technique by Giovanoulis et al^19^ showed findings quite similar to our results with a mean osseous defect of 6.53 cm (SD 15.2) with an average bone union achieved after 13.2 months (SD 7.77), while achieving a consolidation rate of 69.6%.^19^

Hsu et al^20^ published a systematic review and meta-analysis which surprisingly, found that the length of the osseus defect had no significant impact on consolidation rate, but was seen to be associated with a higher infection rate. Similarly, Fung et al^21^ showed the impact of the defect size on the development of postoperative infection. Similar findings about the influence of the defect size have been seen by Griffin et al,^22^ who reported that the therapeutic success rate of the induced membrane technique is independent of the defect size. This affirms the results of a study from Findeisen et al,^23^ where a matched-pair analysis to detect differences between large-sized defects and smaller-sized defects of the lower limb presented a non-significant effect of the defect size on the consolidation rate.

An alternative approach for the treatment of segmental bone defects is distraction osteogenesis using either an external fixator or an intramedullary nail.^24^ This technique offers the principal advantage of eliminating the need for a large bone graft, as required by the Masquelet technique employed in our study. Nonetheless, distraction osteogenesis is not without complications. The literature frequently reports issues such as scarring, adherence of the distraction pins to the surrounding muscle, pain during the distraction phase, and inflammation or infection at the pin sites. Furthermore, nonunion at the docking site, which often necessitates revision surgery, is a recognized challenge.^25^ In June 2024, Wakefield et al^26^ published a systematic review comparing the Masquelet technique (induced membrane technique) with bone regeneration through distraction osteogenesis. This review incorporated data from 32 studies involving 1,136 patients. The authors concluded that there was no significant difference between the two treatment methods, with a union rate of 94.6% for distraction osteogenesis compared to 88.0% for the induced membrane technique. However, there is still a lack of evidence (complete healing with bone remodelling) and further level I studies are to be conducted.^26^

Given the current evidence, age as risk factor for nonunions is controversial. Our results showed no influence of age on the consolidation rate. This confirms other publications in which an insignificant effect of age on the consolidation rate was equally shown.^23,27^ By contrast, Tanner et al^28^ showed that age had a significant influence on the outcome after surgical treatment via Masquelet technique, where patients with successful consolidation averaged out at 66.23 years (SD 6.27), whereas patients without treatment respond presented a mean age of 71.33 (SD 6.00); this is placed in the context of patients aged 58.02 years (SD 14.07) with consolidation and patients without treatment success averaging 59.76 years (SD 12.56) in our results.^28^ Nevertheless, elderly patients are known to be more susceptible to infectious diseases.^29^

Infections are a major complication in the therapeutic process of nonunions. The presence of preoperative infection is identified as a significant factor in the development as well as the persistence of nonunions. Moreover, infection risk increases when presenting larger defects or tibial defects.^21^ This confirms our results, where the consolidation rate in the non-infected collective was 70%, whereas the rate of patients with infection complications was 60%. Even with the advantage of the two-step surgery in the treatment of infected osseous defects, the use of antibiotic-loaded polymethylmethacrylate (PMMA) spacers to achieve an aseptic environment remains contentious. Lu et al^30^ pointed out that the use of antibiotics on spacers did not significantly affect the post-treatment infection or the final consolidation status. This also aligns with previous studies.^20,21^

Smoking has been shown to be a relevant risk factor in prevalence of delayed fracture healing and thus the development of non-unions, by reducing perfusion.^31,32^ Cobb et al^33^ even found for smokers to have a 16-times higher risk for nonunion than non-smokers. In comparison with our descriptive results, ten non-smokers (28.6%) failed to consolidate, but 25 (71.4%) achieved consolidation after two years. Smokers or previous smokers did not show successful nonunion treatment in 40% of patients. However, the subjective smoking status must be viewed critically as many patients give false information, although it is considered reliable in orthopaedic and trauma patients.^34^

The influence of diabetes on developing a nonunion shows no clear trend. Tanner et al^17^ found that diabetes mellitus not only significantly prolonged consolidation time, but also had a significantly negative effect on the therapeutic outcome. Our analysis supports this finding, with five out of ten patients (50%) with diabetes achieved osseous consolidation, whereas of patients without diabetes, 66.7% healed.

Adipositas permagna also poses a further risk factor for nonunion, in addition to many other challenges for orthopaedic and trauma surgeons (stabilization, adequate imaging, or the need for special implants and operative equipment). Kinder et al^35^ published a systematic review about abnormal BMI in orthopaedic trauma patients, in which the prevalence of developing a nonunion for patients with normal BMI (< 30 kg/m^2^) is at 11.1%, while for those with abnormal BMI (> 30 kg/m^2^) it is 16.2%.^35^ Our results do not differ from prior publications, with a failure rate of 56.3% (nine of 15) after two years. Non-obese patients showed a consolidation rate of 70.4%. Comparing the consolidation of treated patients regarding their BMI, the mean BMI of all large-sized defects without osseous healing was around four points higher than the mean index of patients with successful bone healing. Previous publications showed similar results of a negative effect on consolidation rate.^17,36^

According to prior analysis, patients with open fractures have a higher risk of nonunion.^37,38^ This is most likely due to the fact that open fractures are caused by more intensive trauma, resulting in greater destruction of the periosteum and soft-tissue blood supply and generally decreased blood supply, thereby affecting osseous healing.^39^ Moreover, open fractures have a higher probability of local infection, possibly causing further healing complications.^40^ Consistent with these findings, patients with closed fractures had a 10% higher (69.7%) consolidation rate than those with open fractures (59.5%).

This study has a variety of limitations. First, a high number of patients were lost to follow-up, especially at two years. This is probably due to patients presenting from far away, both nationally and internationally. Furthermore, with a long-term follow-up of at least two years, many patients failed to comply. Also, large-sized defects in non-unions of femur and tibia were, thankfully, low in number. Lastly, this study was retrospective; therefore, a randomized trial was not possible.

In conclusion, following the treatment of large-sized nonunions of the lower limb, a structured and sufficiently long follow-up is required to secure the consolidation status. At least two years of follow-up are needed to reach full consolidation (Lane-Sandhu score > 2), as a significant number of patients healed within the second year after surgical treatment. Furthermore, the preferred treatment via two-step procedure (induced membrane technique) seems to be a reliable and effective solution in surgical therapy of lower limb nonunions with defects of all sizes.

**Take home message**

- A follow-up of two years should be awaited before considering a revision surgery.

- Furthermore, a staged approach for infection control and improvement of local circulation seems reasonable.

## Data Availability

The data that support the findings for this study are available to other researchers from the corresponding author upon reasonable request.
